# Epilepsy Websites in Africa: State of the Art

**DOI:** 10.7759/cureus.15224

**Published:** 2021-05-25

**Authors:** Yahya Naji, Tawab Ait Abdelmoula, Khawla Ait Mhand, Klevor Raymond, Najib Kissani

**Affiliations:** 1 Neurology Department, University Hospital Mohammed VI, Marrakech, MAR; 2 Neurology Department, Medical Research Center, Marrakech Medical School, Cadi Ayyad University, Marrakech, MAR

**Keywords:** epilepsy, websites, africa, education, awareness

## Abstract

Purpose

Epilepsy is one of the main brain disorders in the world and should be considered a priority for healthcare in Africa. Internet is a growing source of health information for healthcare and patients with epilepsy (PWEs). In this short communication, we tried to describe the status of websites on epilepsy in Africa in terms of availability and pertinence, and to suggest ideas on how to make them more effective*.*

Methods

The existence of epilepsy websites and their distribution in Africa were acquired using a questionnaire sent to different neurologists in the African continent.

Results

Based on the survey answers and the web search, our results show that out of 54 African countries, only 16 countries (≈30%) have an active epilepsy website.

Conclusion

The need for reliable, clearly written, and easily comprehended information about epilepsy is considered as an important element to enhance the quality of care, for this all African countries should create and improve their epilepsy websites in order to promote education and awareness.

## Introduction

Epilepsy is one of the most common and serious brain disorders in the world. It affects people of all ages and is characterized by unpredictable recurrent seizures [1]. The African region is composed of 54 countries with a total population of 1,308,064,000 [2]. Epilepsy affects 10 million* *peoples of all ages, but especially within childhood, adolescence, and the aging population [1]. Prevalence rates in the African region range from 2.2 to 58 per 1000, while risk factors are dominated by poor perinatal care, head trauma, and intracranial infection [1].

Epilepsy is not only a medical condition; it also includes sociological and cultural dimensions. Africa has more than 24% of all illiterate adults in the world [3]. So it is common for people in many African countries to see epilepsy as the manifestation of a supernatural force. Consequently, many of these patients and their families first consult traditional healers and follow their recommendations for a long period of time [4].

The high rate of illiteracy and the lack of resources within the health system with a low doctors/population ratio are factors that allow epilepsy to affect the physical, psychological, and social functioning of patients and their families [5]. In order to combat the false view of epilepsy and with the explosive growth of internet coverage in Africa, websites seem to be a very interesting way to communicate medical information and raise awareness.

## Materials and methods

To obtain information on the existence of websites specializing in epilepsy and their contribution in African countries, a questionnaire was designed using a Google Forms survey application. The questionnaire was displayed on a single webpage with a submit button at the end of the page. The Google Forms uniform resource locator (URL) was distributed as a link by email sent to all neurologists in the African countries. Respondents entered and submitted answers directly via Google Forms URL. Each respondent was restricted to only one possible response in order to avoid duplicate response errors. Information collected includes the title, the country, and the number of neurologists, affiliation to the International Bureau for Epilepsy (IBE) and International League Against Epilepsy (ILAE), and the existence of epilepsy website.

The questionnaire was distributed between March 2020 and August 2020. Data were stored by Google Forms. About the countries for which we didn’t get answers by the questionnaire, we proceed to a web search using Google search engine, which is the most used search engine in Africa. The keyword combinations used were the epilepsy website and each African country (in French and English). The list of web pages was included if they give educational, medical, or health information about any aspect of epilepsy. The results of the web research which does not serve to achieve our objectives have been eliminated. Statistical analyses were carried out with the software Microsoft Excel for Windows version 2007.

## Results

**Figure 1:**
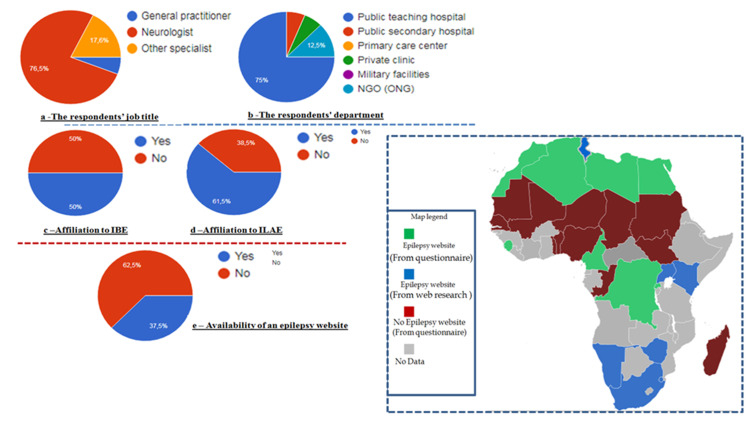
At left: The analysis of questionnaire responses include information about the respondent, the availability of an epilepsy website, the affiliation to the International League Against Epilepsy (ILAE) and International Bureau for Epilepsy (IBE) / At right: Map showing all the African countries with or without data on the existence of website epilepsy (in green we found the results from the questionnaire, in blue we show the results from the web research, in red we display countries without website based on the survey, while in grey we show countries without any data concerning the presence or not of an epilepsy website.

The questionnaire was sent to more than 54 neurologists from 33 different African countries. A total of 27 answer forms from 22 different countries were received (≈41 % of all the African countries). The response rate varied substantially between different countries. The analysis of the questionnaire results found that the majority of respondents were neurologists in 76%, mainly working in public teaching hospitals (77%). From the 22 countries, the affiliation to ILAE was over 61%, while affiliation to IBE was about 50% (Figure 1). The existence of epilepsy website based on the questionnaire answers was less than 35% (about nine countries), the majority of this website was created in the last five years (≥2015) (Table 1). For the 25 countries in which we didn’t get answers, we proceed to a web search using the Google search engine for the availability of epilepsy websites. We get seven more African countries with functional and educative websites dedicated to epilepsy (Table 1). Through our study, we made a map showing all the African countries with or without data on the existence of website epilepsy (Table 1). 

**Table 1:** Availability of website in the African countries.

Country (ies)	Answers (number)	Epilepsy website link	Year of creation
Algeria	1	www.lape.dz	2018
Benin	1	None	-
Cameroon	1	www.codefcameroon.wordpress.com	2018
Chad	2	None	-
Congo-Brazzaville	1	None	-
Democratic Republic of Congo (DRC)	1	http://www.aslek.org	2018
Egypt	1	https://www.facebook.com/وحدة-اضطراب-كهربية-المخ-قصر-العيني-جامعة-القاهرة-1694738570799460/1	2019
Gambia	1	None	-
Libya	2	https://www.facebook.com/aoaunitepilepsy	2010
Madagascar	1	None	-
Mali	1	None	-
Mauritania	1	None	-
Mauritius	1	http://edycs.org	2017
Morocco	3	www.neuromarrakech.com www.dimaghy.com	2015 2021
Niger	1	None	-
Nigeria	2	None	-
Rwanda	1	www.gecorwanda.org	2018
Senegal	1	None	-
Sierra Leone	1	www.epilepsyassocsl.org	2010
South Sudan	1	None	-
Sudan	1	None	-
Togo	1	None	-
Kenya	None	www.kawe-kenya.org	-
Namibia	None	www.epilepsynamibia.org	-
South Africa	None	www.epilepsy.org.za	-
Swaziland (Eswatini)	None	www.epilepsy.org.sz	-
Tunisia	None	https://www.facebook.com/epilepsietunisie/	-
Uganda	None	www.epilepsy.org.ug www.purplebenchug.org	-
Zimbabwe	None	https://www.facebook.com/epilepsysupportzim/	-

Our results show that out of 54 African countries, only 16 countries (≈30%) have an epilepsy website. While some countries excel in virtual epilepsy education and support, others don’t even own a local center or association for this matter, let alone a website, despite the high prevalence of this disease among African countries. Countries of North and South Africa come first in the availability of epilepsy websites compared to other regions of the African continent. Morocco, Algeria, Namibia, Swaziland, and South Africa have specific websites, dedicated to improving the epilepsy practice field and its neurophysiologic exploration. As for Egypt, Libya, and Tunisia, they have Facebook pages where they share useful information and updates with their followers. While Cameroon and the Democratic Republic of the Congo are an example for the countries of Central Africa which have an online platform; where people can get all information and instructions regarding epilepsy and its different aspects. They even pay home visits and share inspiring stories despite the huge pressure these countries are facing. In the East part of Africa, Kenya and Uganda established an online source, where they strongly defend quality care and equal opportunities, as well as empowering patients with epilepsy (PWEs) through awareness and second chances, by disseminating correct medical explanations and eradicating misconceptions.

## Discussion

Epilepsy is the fourth most common neurological disorder worldwide [6]. It affects people of all ages and is characterized by unpredictable seizures [1]. Patients and their families should learn as much as possible about types of seizures, appropriate management, safety, and quality of life. Recently, there has been an explosive growth of the internet and websites as tools for seeking and communicating health and medical information. Public knowledge and attitudes towards epilepsy have been repeatedly investigated in developed and developing countries. Large gaps have been found in awareness of the causes of the disease, and, even worse, widespread negative attitudes were documented, mainly associated with educational level, age, and gender [4].

The results revealed that 2/3 of the African countries don’t have web pages dedicated to epilepsy. This lead to many African patients claiming information from incredible sources. The low number of epilepsy websites in Africa besides other socioeconomic and healthcare factors, keeps epilepsy greatly misunderstood, deeply stigmatized, severely underfunded, and most often, ignored by the healthcare system. Relatively some web pages displayed non-scientific information, while more accurate information was provided by websites of medical institutions and general health websites.

With changing times, people are becoming more and more aware of the need to seek scientifically founded information. The world today is too conscious of the impact of epilepsy, but many people in African countries keep a sort of transition between non-scientific and scientific practices. This transition does not automatically embrace the full tenets of medical understanding. It appears that even with the presence of websites in some countries, the rate of epileptic patients properly treated is very low. It can be expounded by either the malfunctioning of the site, the inadequate and disproportionate proposal of the information within the website, or the socio-economic status of the African population. For that not only websites should be built; they must contain simple and significant information serving as a guide to people in need. Furthermore, websites should be supervised by the local health authority centers. These centers are obliged to check the utility and the value of giving information in order to build trusted websites concurrently with reputed sources. Websites alone aren’t enough, but the use of full technological advancements could help the African continent to improve medical knowledge and care to underserved populations.

## Conclusions

Despite the efforts made by the African countries in the fight against epilepsy, they should expand more South-South collaborations, along with establishing local African guidelines for epilepsy management and advocacy through websites and telecommunication ways. Developing electronic platforms with databases in Africa will promote epilepsy care and provide credible sources.
